# Epilepsy and chromosomal abnormalities

**DOI:** 10.1186/1824-7288-36-36

**Published:** 2010-05-03

**Authors:** Giovanni Sorge, Anna Sorge

**Affiliations:** 1 Department of Pediatrics, Azienda Ospedaliera Universitaria "Policlinico - Vittorio Emanuele", Università di Catania, Via Santa Sofia 78, Catania 95123, Italy

## Abstract

**Background:**

Many chromosomal abnormalities are associated with Central Nervous System (CNS) malformations and other neurological alterations, among which seizures and epilepsy. Some of these show a peculiar epileptic and EEG pattern. We describe some epileptic syndromes frequently reported in chromosomal disorders.

**Methods:**

Detailed clinical assessment, electrophysiological studies, survey of the literature.

**Results:**

In some of these congenital syndromes the clinical presentation and EEG anomalies seems to be quite typical, in others the manifestations appear aspecific and no strictly linked with the chromosomal imbalance. The onset of seizures is often during the neonatal period of the infancy.

**Conclusions:**

A better characterization of the electro clinical patterns associated with specific chromosomal aberrations could give us a valuable key in the identification of epilepsy susceptibility of some chromosomal loci, using the new advances in molecular cytogenetics techniques - such as fluorescent in situ hybridization (FISH), subtelomeric analysis and CGH (comparative genomic hybridization) microarray. However further studies are needed to understand the mechanism of epilepsy associated with chromosomal abnormalities.

## Background

Many chromosomal abnormalities are associated with Central Nervous System (CNS) malformations and other neurological alterations resulting in mental retardation (MR) and seizures that are more frequent than in general population [1-3]. Certain chromosomal syndromes are specifically associated with epilepsy and show a particular clinical and EEG pattern such as 1p36 monosomy, Wolf-Hirschhorn syndrome, ring 20 chromosome syndrome, Miller-Dieker syndrome, 18q- syndrome, and Down syndrome. Other congenital anomalies, due to chromosomal imbalance, differently from the above mentioned, have no specific patterns of seizures even if these are frequent, for example the 14r syndrome, the Klinefelter syndrome, the Fragile-X syndrome. Some of these will be described successively.

## Results and Discussion

For all these syndromes a better knowledge of the electro clinical patterns associated with specific chromosomal aberrations could give us a valuable key in the identification of epilepsy susceptibility of some chromosomal loci and the new advances in molecular cytogenetics techniques such as fluorescent in situ hybridization (FISH), subtelomeric analysis and CGH (comparative genomic hybridization) microarray might allow it [4].

### 1p36 Monosomy

The 1p36 monosomy is a syndrome quite recently recognized and characterized by mental retardation and multiple congenital anomalies [5-7]. The estimated incidence is 1/5000 to 1/10000 births [8]. The affected patients show a peculiar phenotype consisting in microcephaly, brachycephaly, large and late-closing anterior fontanels, prominent forehead, straight eyebrows, short palpebral fissures, deep-set eyes, flat nasal bridge, midface hypoplasia, elongated philtrum, pointed chin, hypotonic face, low-set malformed and posterior rotated ears. Moreover patients show brachydactyly of the fifth finger, camptodactyly, short feet, hearing loss mostly of the sensorineural type, various skeletal anomalies, urogenital anomalies, congenital heart defects, cardiomyopathy, muscle hypotonia, congenital hypothyroidism, different degree of cognitive impairment varying from severe to moderate with poor or absent language. Seizures are present in 50-58% of patients. Age of seizure onset is not well defined, but generally they start in infancy or childhood [9-11]. The seizures are of different types including infantile spasms, generalized tonic-clonic seizures, complex or single partial seizures, myoclonic seizures and absence seizures. Their control with current antiepileptic drugs is quite good. The spectrum of EEG abnormalities varies widely and includes hypsarrhythmia, focal and multifocal spikes and asymmetry of slow wave activity. Infantile spasms are the most frequent type of seizures (25% of patients) and, according to the literature, are well controlled by corticotropin in the majority of the cases [7]. All kind of seizures seem to improve with time and only few cases of intractable epilepsy are reported. However the epilepsy is highly variable among the affected patients [11]. Most common finding at the brain magnetic resonance imaging (MRI) is cortical atrophy with enlargement of the lateral ventricles. Some patients show non specific posterior white matter abnormalities or delayed myelination. In some case a thin corpus callosum is described [9,12,13]. The causes of epilepsy in 1p36 monosomy remain unknown. The literature suggests that the characteristic phenotype varies with the size of the deletion being more pronounced in larger deletions. The terminal region of chromosome 1p is particularly gene-rich. Among the genes present in this area, two of them might account for epilepsy: KCNAB2 or potassium channel-β subunit gene and GABRD or γ-aminobutyric acid A receptor δ subunit gene. Regarding the KCNAB2, it was hypothesized that its haplo-insufficiency could decrease the threshold for seizures, but this hypothesis is in contrast with the observation of patients with 1p36 deletion and intractable epilepsy in which no loss of KCNAB2 was present. Also the GABRD gene maps on 1p36, but until now, no correlation with the seizures has been demonstrated [7,14,15].

### Wolf-Hirschhorn syndrome (4p- syndrome)

Wolf-Hirschhorn syndrome, a well known clinical entity, is caused by a partial deletion of the short arm of chromosome 4. Its frequency is estimated as one per 50000 births with a female predilection of 2:1 [16-18]. The syndrome is characterized by typical cranio-facial anomalies consisting of microcephaly, ocular hypertelorism, epicanthic folds, coloboma of the iris, prominent glabella with a "Greek warrior elmet" appearance, cleft lip and/or palate, severe mental retardation and high prevalence of seizures (80-90%) that include alternate hemiconvulsion, febrile seizures, infantile spasms frequently drug resistant [19-21]. The mean age at the seizure onset is 9 months ranging from 5 to 23 months [22,23], but some patients are reported with neonatal onset of generalized seizures accompanied by EEG abnormalities [20,21]. Many patients with resistant status epilepticus are also reported. Distinctive EEG patterns are observed in about 70% of patients [21]. These patterns are essentially of two types: one is characterized by frequent, diffuse, atypical slow sharp element, spike and wave complexes often occurring in long bursts activated by slow wave sleep, with paroxysmal activity associated with typical absences. The second type shows frequent high amplitude, fast spikes-polyspikes and wave complexes over the posterior third of the head, triggered by eye closure [11,24-26]. These abnormalities are even present in patients who had not experienced seizures [20-23]. Epilepsy is generally well-controlled with monotherapy alone and there is good evidence of general improvement of seizures with age [21]. At brain Magnetic Resonance Imaging (MRI) the most frequent anomaly is corpus callosum hypoplasia together with agenesis or hypoplasia of posterior lobe of cerebellum. Heterotopias and dysplastic nuclear structures are also reported [4].

### Angelman syndrome

Angelman syndrome can result from several mechanisms that lead to the loss of maternally imprinted gene function. Deletions within bands 15q11-q13 of the maternally derived chromosome is present in 70% of cases. Paternal uniparental disomy is found in about 2-5% of cases; remaining cases are presumed due to a mutation in a specific gene. According to literature, patients with the chromosomal deletion have a more severe phenotype than those with uniparental disomy form or those with the single gene anomaly [19,27-29]. The clinical phenotype is characterized by microcephaly, frontal upsweep, prominent mandible, pointed chin, protruding tongue, diffuse depigmentation. Moreover patients show severe mental retardation, inappropriate laughter, happy disposition, ataxic gait, jerky movements, spasticity, lack of speech [30]. Seizures occur in 80-90% of patients and the onset is usually between 1 and 2 years, but seizures can occur in infants less than 1 year old or older than 3 years [31]. Atypical absences and myoclonic seizures are common as well as non convulsive status epilepticus. Different seizures types can occur in each patient. Hyperkinetic stereotypes and behavioural disturbances are at risk to be misinterpreted and considered epileptic manifestations leading to unjustified overtreatment. Often the convulsions are refractory to therapy. EEG is characteristic and demonstrates two types of epileptic discharge: burst of high-voltage frontal dominant activity (2-3 c/s) associated with spikes and sharp waves and bursts of occipital dominant high-voltage activity (3-4 c/s) associated with spikes [32].

### Miller-Dieker syndrome

Miller-Dieker syndrome is associated with microdeletions within bands 17p13.3 that includes the LIS1 gene. The syndrome is characterized by classic lissencephaly or type 1 lissencephaly, due to a neuronal migrational arrest between 12 and 16 weeks of gestation resulting in a cortex with four instead of six layer, profound mental retardation, hypotonia and spasticity, epilepsy and typical facial features consisting of prominent forehead, bitemporal hollowing, short nose with upturned nares, protruding upper lip, thin vermillion border of upper lip and small jaw. Seizures, generally drug-resistant, are precocious and occur very often in the first months of life in form of partial, tonic, myoclonic or infantile spasms in 90% of cases [19,33-35]. The EEG is characterized by high-amplitude α or β activity alternating with high amplitude slow rhythms and simulates slow spike-wave complexes or hypsarrhythmia. The disease follows a grim course with only some patients surviving into adult life [19].

### 18q- syndrome

Several reports suggest the association of epilepsy and 18q- syndrome [3]. This chromosomal aberration is associated with various dysmorphisms consisting in microcephaly, turricephaly, deep-set-eyes, carp shaped mouth, broad nasal bridge, high arched or cleft palate, small hands and feet, frequent cardiac anomalies. Patients show moderate to severe mental retardation, aggressive behaviour, ataxia, dysmetria, and hypotonia [29]. Disturbed myelination pattern with cerebellar hypoplasia and hydrocephalus are often present. Early epilepsy can be a feature of the syndrome [36]. The reported seizures are frequently autonomic epileptic seizures with cardiac arrhythmia and apnoea simulating non epileptic syncope [7,36,37].

### Ring chromosome 20 syndrome

Ring chromosome 20 syndrome has a striking association with epilepsy whereas in the other aberrations of the same chromosome epilepsy is rarely reported [19]. Generally a chromosomal mosaicism is present [38]. The syndrome is not characterized by a particular phenotype instead seizures occur virtually in all cases. The reported phenotypic anomalies are represented by microcephaly, facial dysmorphism with hypertelorism, high arched palate, long neck and trunk, thenar and hypothenar hypoplasia, microgenitalism, tetraparesis, dysarthria and sever MR, even though in many reports the intellect is described within the normal range or mildly deficient [19,39]. Characteristically there is a worsening of clinical and EEG features over a long period of time and, for this reason, an early diagnosis is often difficult [19]. Age at onset of seizures varies from infancy to 14 years and the seizures are characteristically resistant to treatment [40-45]. According to the data published by Ville et al [39] the epilepsy seems to have particular patterns that consist of the following:

• a normal or nearly normal EEG activity with inconstant runs of theta waves in fronto-central areas not significantly influenced by eye opening, level of vigilance or intravenous injection of diazepam;

• in other instances episodes of nonconvulsive status epilepticus with a prolonged confusion and, on the EEG, long-lasting high-voltage slow waves with occasional spikes usually frontal, sometimes unilateral are observed. Spike-and-wave complexes are not a predominant feature. Otherwise there is the presence of focal seizures associated to ictal terror and hallucinations with loss of consciousness, oro-alimentary automatisms and hypertonia with frontal onset discharges of short duration;

• pharmacoresistance;

• psychomotor delay or major behavioural disturbances [39].

As before mentioned, the first seizure often occurs in childhood, but most of the reported patients are adolescents or adults probably because the time lag from onset to diagnosis is usually long after many useless clinical investigations and ineffective treatments and, in many cases, psychiatric treatment was administered for several years without any improvement [39]. For this reason a cytogenetic study should be performed on all patients having epilepsy, dysmorphic features and/or learning disabilities [44]. Some cases are reported with a neonatal onset of the epilepsy [46-50]. In these patients seizures were generalized and the EEG was normal or showed diffuse slowing. It was suggested that the severity of clinical features may depend on the extent of chromosomal deletion [45] or on the entity of lymphocyte mosaicism [38].

### Down syndrome

**Figure 1:**
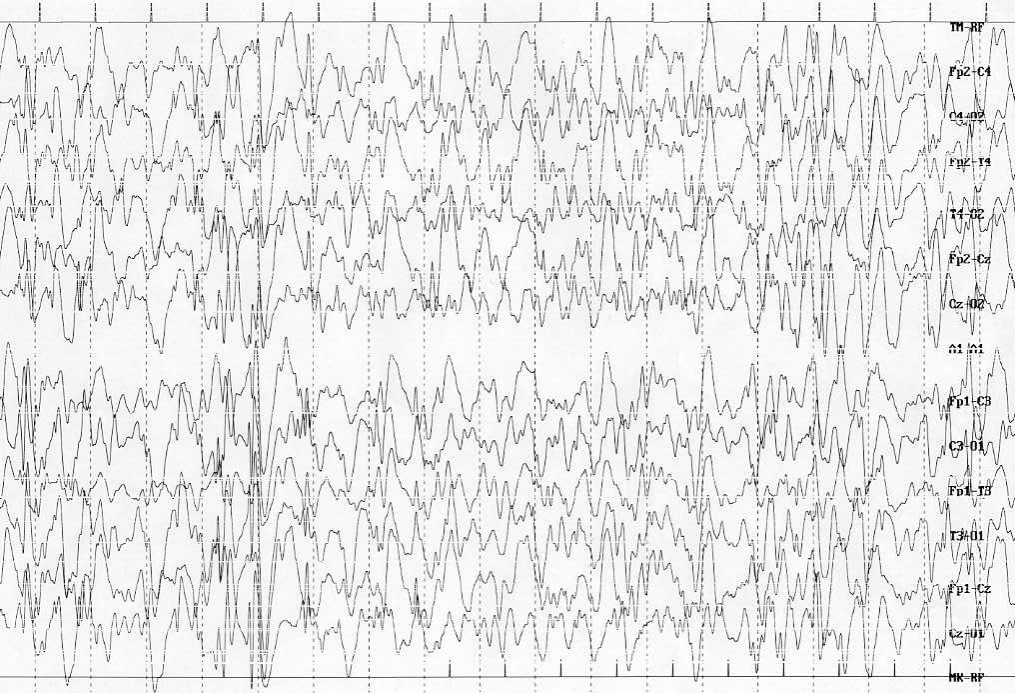
**Down Syndrome: Infantile spasms - hypsarrythmia**.

Epilepsy occurs in 8% of individuals with Down syndrome (DS). Age of seizure onset is bimodal: 40% occurs before 1 year of age and 40% occur in the third decade of life [51]. However epilepsy in DS is less common than in most mental retardation syndromes (19). The increased seizure susceptibility has been attributed to inherent structural anomalies of the brain [52-54] such as fewer inhibitory interneurons, decreased neuronal density, abnormal neuronal lamination, persistence of dendrites with foetal morphology or primitive synaptic profiles [55,56]. In patients with DS has been also documented an altered membrane potassium permeability that increases the voltage threshold for spike formation, reduces hyperpolarization following spikes or increases action potential duration [53-55]. Some authors report that seizures in DS could be associated with medical complications such as cardiovascular abnormalities, recurrent infections or nutritional deficiencies [56-60]. All major seizure types have been described in children with DS: 47% of patients develop partial seizures, 37% infantile spasms and 21% generalized tonic-clonic seizures [54,61]. In the younger age the predominant type of convulsions are represented by infantile spasms and tonic-clonic seizures with myoclonus [61]. Infantile spasms, particularly frequent in male patients, are usually associated with a poor long-term prognosis (Fig. 1) [19,51,62-65]. However about half of the children with infantile spasms achieve seizure remission without relapse and partial restoration of development. In the other 50% who still have seizures, there is no difference in outcome between groups treated with valproic acid, cotricotropin, or both [51-54]. When seizures occur during the childhood or in the third decade of life, generalized tonic-clonic are the most common together with partial simplex or partial complex seizures, but myoclonic, atonic and absence with tonic-seizures have been reported [19,61].

### Other syndromes

In many other chromosomal disorders, seizures, not as typical as in the above-mentioned syndromes, can be found.

Convulsions are reported in patients affected with deletion of the long arm of chromosome 1 (**1q- syndrome**). In this syndrome, characterized by microcephaly, severe MR, high-pitched cry, abnormal posturing, agenesis of corpus callosum and diaphragmatic hernia, seizures usually begin in the first 3 years of life. Various types of seizures are reported: generalized, febrile, complex partial or not well defined [19,66-68]. All types of seizures are often well controlled, but we do not have data about the long-term outcome of epilepsy. The EEG patterns reported in literature are described as multifocal or bilateral central epileptiform discharge, or as centro-temporal spikes similar to rolandic spikes [19,69].

Seizures are also reported in patients with chromosome 2 abnormalities. Regarding **2p deletion syndrome **there are isolated cases of generalized or febrile or myoclonic seizures with onset between 6 months and 2 years of life. The clinical phenotype of these patients is characterized by microcephaly, severe MR and a facial resemblance to Down syndrome [19]. The reported EEG abnormalities consist in focal epileptic form or generalized spike-waves discharges [70].

In patients with **2q deletion syndrome **severe forms of early onset epilepsy consisting in status epilepticus, myoclonic seizures, generalized seizures and drugs resistant seizures, have been reported [3,19,71] even if convulsions are not the prominent feature. In these patients various patterns of EEG are reported: generalized and centro-temporal spikes, slow background or centro-temporal-frontal spikes and waves [71].

Seizures with fever are reported in the patients affected with the rare **short arm of chromosome 3 deletions syndrome **in which MRI abnormalities are very severe [19].

There are no deletion or duplication syndromes associated with epilepsy on the short arm of chromosome 5 [19].

In 25% of patients affected with **distal deletion of the long arm of chromosome 6 **seizures, with age of onset ranging from 4 months to 10 years of life, are described. Most are generalized convulsions, but infantile spasms or complex partial seizures are also reported [72]. Elia et. al. [73] reported 5 patients with a peculiar clinical and EEG pattern constituted by early, focal epilepsy originating from the occipital lobes in most cases associated with heterotopia or neuronal migration defects, colpocephaly and dysgenesis of the corpus callosum, thalami and brainstem.

Seizures onset ranging from neonatal period to 7 years are reported in patients affected with **deletion of the long arm of chromosome 7**. Febrile, generalized, myoclonic and combination of afebrile and febrile seizures are described in these cases [19]. All patients show MR and microcephaly. EEG studies are mentioned only in a few cases and the detected anomalies consist in hypsarrythmia, rolandic spikes and multifocal spikes [19].

Seizures occur in <10% of cases of **deletion of the short arm of chromosome 9 **[19,74]. Generally the patients have complex partial seizures and the clinical phenotype is characterized by short stature, MR, microcephaly and severe kyphosis.

There are only isolated case reports described with epilepsy on chromosome 10 and 11 [3,19,75].

**Trisomy 12p **is frequently associated with myoclonic absence [76]. It is a syndrome characterized by MR, characteristic cranio-facial anomalies consisting in high forehead, long face, succulent periorbital tissue, downslanting palpebral fissures, flat nasal bridge, high palate, limbs deformity such as camptodactyly of both hands and feet, poor sucking and congenital heart defects [77].

The **Pallister-Killian syndrome **a rare, sporadic disorder caused by a mosaic supernumerary chromosome 12p [78], has been associated with West syndrome [76].

**Figure 2:**
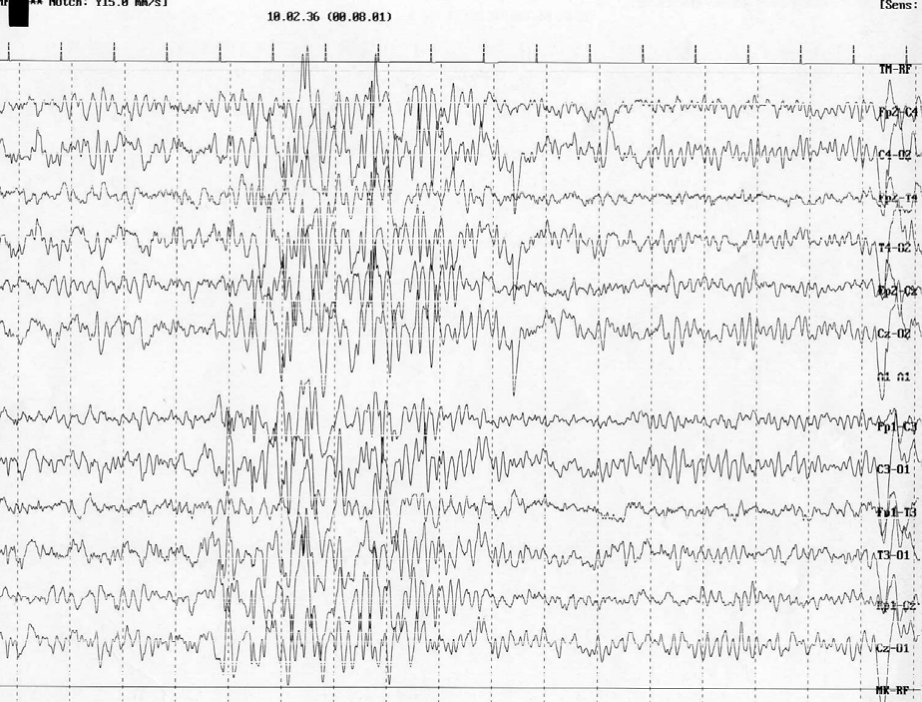
**Trisomy 13: Generalized seizures - Centro-temporal-frontal spikes and waves**.

In **trisomy 13 **or Patau syndrome seizures are reported rarely even if a variety of developmental abnormalities of the brain are present: holoprosencephaly (60-80% of cases), cerebellar dysplastic changes, olfactory aplasia, hippocampal hypoplasia and callosal agenesis. In most cases, seizures develop during the neonatal period. Most are multifocal clonic or photosensitive myoclonic type (Fig. 2) [75-80]. Due to severe multiple congenital anomalies the mortality is > 80% within the first month of life.

The only anomaly of chromosome 14 with a striking association with epilepsy is the **ring 14**. Numerous cases are reported, some of which familial [19,81,82]. Generally seizures begin in infancy and various types have been described: generalized tonic-clonic seizures, myoclonic, minor motor seizures, complex partial seizures [82-84]. The clinical spectrum is characterized by generalized hypotonia, ataxia, retinal and macular abnormalities, cerebral atrophy and ventricular dilatation.

Recently reported is a syndrome due to **15q13.3 microdeletion **associated with mental retardation, developmental delay and seizures [85]. In these patients, seizures were of various types: myoclonic seizures, absence seizures, tonic-clonic seizures, intractable epilepsy [85]. Affected patients show everted lips, deep-set eyes, upslanting palpebral fissures, hypertelorism, synophris, prominent philtrum, and hypotonic facies.

Epilepsy is an uncommon manifestation associated with **22q11 deletion**. It is present in less than 5% of the patients [86,87].

The estimated incidence of epileptic manifestations occurring in association with **Klinefelter syndrome **is 5-17% [88]. The age of onset of seizures is within 3 months and 3 years of age. Febrile seizures, generalized tonic-clonic seizures, complex partial seizures and absence seizures are reported [89,90]. EEG abnormalities consist of epileptiform discharges, typically focal or multifocal. The patients with Klinefelter syndrome and epilepsy frequently have some degree of mental handicap and behaviour difficulties. Neuro-imaging studies failed to identify structural basis of seizures [90].

**Figure 3:**
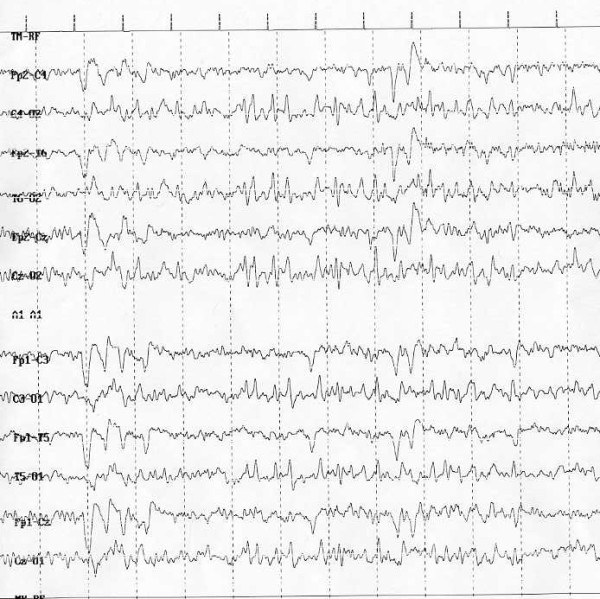
**Fragile-X: Occipital spikes and waves**.

**Fragile-X syndrome **(Fra-X) is the most frequent cause of familial MR and is the second most common cause of mental disability after DS [91]. Epilepsy in patients Fra-X syndrome was first reported in 1969 [92]. The frequency of epilepsy is reported from 13-14% to 41-44% [93,94]. Types of seizures in Fra-X show similarities with some epileptic syndromes such as benign childhood epilepsy with centro-temporal spikes, childhood epilepsy with occipital paroxysms (Fig. 3), partial motor seizures, Landau-Kleffner syndrome, partial frontal epilepsy with favourable evolution and status epilepticus during sleep [91,95]. EEG anomalies similar to the benign childhood epilepsy with centro-temporal spikes (BCECTS) are frequently reported [91,93,95,96]. Incorpora et al. [91] proposed that the Fra-X cases reported in her paper could be grouped according to the pattern of seizures: with normal EEG and no seizures, with EEG abnormalities without seizures, with EEG abnormalities and well-controlled seizures and with EEG abnormalities and severe seizures unresponsive to treatment. Usually both epilepsy and EEG improve with age.

## Conclusions

Epileptic syndromes are frequently reported in chromosomal disorder. In some of these congenital syndromes the clinical presentation and EEG anomalies seem to be quite typical, in others the manifestations appear nonspecific and not strictly linked with the chromosomal imbalance. Very often the onset of seizures is during the neonatal period or the infancy. Further studies are needed to well delineate the clinical features of epileptic syndromes and to understand the mechanisms of epilepsy associated with chromosomal abnormalities.

## List of abbreviations

CNS: Central Nervous System; MR: Mental Retardation; FISH: Fluorescence In Situ Hybridization; CGH: Comparative Genomic Hybridization; MRI: Magnetic Resonance Imaging; DS: Down's Syndrome.

## Competing interests

The authors declare that they have no competing interests.

## Authors' contributions

GS conceived, designed and coordinated the study, AS participated in literature and ichnographic research
